# Towards evidence-based management of patients treated with cardiotoxic chemotherapy: A collaborative effort of cardiologists and oncologists

**DOI:** 10.1007/s12471-018-1163-8

**Published:** 2018-10-01

**Authors:** J. M. Leerink, Y. M. Pinto

**Affiliations:** Department of Clinical and Experimental Cardiology, Amsterdam Cardiovascular Sciences, Amsterdam UMC, University of Amsterdam, Amsterdam, The Netherlands

Chemotherapeutic agents have contributed importantly to the increased survival of patients with cancer. However, with greater survival, their cardiotoxic side effects emerge more often as a source of great concern [1]. Already in the 1970s during the first trials with anthracyclines, a potent antineoplastic agent, patients died due to congestive heart failure [2–4] which was, with the current knowledge, likely to be caused by the toxic effects of anthracyclines on cardiomyocytes which led to a decreased left ventricular function. However, almost 50 years after their first routine use, the exact mechanism by which anthracyclines induce cardiotoxicity remains elusive [5].

In addition to anthracyclines, several other chemotherapeutic agents that were used successfully to treat a wide variety of cancers were found to be toxic for the heart. Potential cardiotoxic side effects of antineoplastic agents include but are not limited to myocardial dysfunction, arrhythmias, ischaemia, myocarditis and pericarditis [6].

Knowledge of these cardiotoxic side effects and the increased survival of patients with cancer has urged cardiologists and oncologists to identify risk factors (e. g. higher cumulative dose), preventive measures (e. g. dexrazoxane), early detection tools and treatment options to reduce the risk of chemotherapy-induced heart failure. This led to a position paper on cancer treatments and cardiovascular toxicity by the European Society of Cardiology in 2016 [7]. This position paper discusses management, prevention and treatment of cardiovascular complications of different cancer therapies. However, due to a lack of evidence in the cardio-oncology field it lacks uniform recommendations for management of patients around treatment with cardiotoxic chemotherapies.

In the current issue of the Netherlands Heart Journal, Teske and colleagues give an inspiring overview on the management of oncology patients treated with cardiotoxic chemotherapies at their cardio-oncology outpatient clinic, which stems from a close collaboration between oncologists and cardiologists [8]. In contrast to the above-mentioned position paper they provide clinicians with a practical and useful strategy for the management of these patients. The authors use a score slightly modified from that of Hermann et al. [9] for risk estimation of cardiac dysfunction before chemotherapy initiation, which incorporates type of chemotherapeutic agent and patient characteristics to decide on the need and frequency of cardiac surveillance during and after oncology treatment. Furthermore, they discuss the usefulness of different surveillance modalities in different stadia during and after cancer treatment and advise on indications for initiation of heart failure medications.

However, as the authors also mention, evidence for their management strategy remains scarce and mainly comes from studies on anthracycline and/or trastuzumab cardiotoxicity and leaves room for discussion. The overview advises echocardiographic surveillance for up to 1 year after chemotherapy cessation in adult patients based on a study by Cardinale et al. showing that development of left ventricular dysfunction beyond 1 year is rare [10]. This contrasts with childhood cancer survivors who are known to be at long-term risk of heart failure [11] and are therefore referred to the Dutch long-term effects after childhood cancer outpatient clinic (LATER clinic) [12]. Although evidence from Cardinale et al. seems promising we would like to advocate surveillance of cardiac function beyond 1 year after chemotherapy cessation in adults, especially in high-risk patients, until more studies confirm the safety of a single year follow-up strategy and more accurate risk prediction is possible.

Also, the ejection fraction (EF) thresholds (EF <45% or >10 point EF decline <53% and NYHA II/IV) used for initiation of heart failure medications are based on two non-randomised trials and require more studies to support or refine these thresholds.

Nonetheless, this overview from Teske and colleagues provides a useful framework for the management of patients treated with cardiotoxic chemotherapy and this should serve as an incentive for more research on mechanisms, risk estimation, early detection, prevention and treatment of chemotherapy-related cardiac dysfunction. All of which would greatly benefit from close collaboration between cardiologists and oncologists.
